# Exploring the Associations Between Autistic Traits, Sleep Quality, and Well-Being in University Students: A Cross-Sectional Study

**DOI:** 10.3390/brainsci15060567

**Published:** 2025-05-25

**Authors:** Devangi Lunia, Andrew P. Smith

**Affiliations:** Centre for Occupational and Health Psychology, School of Psychology, Cardiff University, 63 Park Place, Cardiff CF10 3AS, UK; smithap@cardiff.ac.uk

**Keywords:** autistic traits, broader autism phenotype, sleep quality, well-being, university students, depression, anxiety

## Abstract

Background: Very few studies have examined associations between autistic traits, sleep, and the well-being of university students, and the aim of the present study was to address this knowledge gap. Methods: Three hundred and eight university students carried out an online survey consisting of the Comprehensive Autistic Traits Inventory (CATI), the Short-Form Well-Being Process Questionnaire (SWBPQ), the Short-Form Strengths and Difficulties Questionnaire (SDQ), and the Short-Form Sleep Questionnaire. Results: Univariate analyses revealed significant correlations between the CATI subscales and the SDQ outcomes, but there were few associations between the CATI scales and the well-being outcomes, which were associated with the sleep scores and the well-being predictors. No interactions were found between the predictor variables. This profile was confirmed in the multivariate analyses, which also showed significant associations between the CATI scores and well-being predictors. Conclusions: CATI scores were associated with the outcomes of strengths and difficulties but showed few significant associations with the WPQ outcomes, which were predicted by sleep and well-being predictors. However, evidence of the indirect effects of the CATI scores on well-being came from the associations between the CATI scales and the well-being predictors.

## 1. Introduction

The research described in this article aimed to extend our knowledge of autistic traits, sleep, and well-being, adopting approaches that are novel in the area. The first feature of the research was a focus on autistic traits rather than diagnosed individuals. Well-being was measured using an approach that assesses both positive and negative outcomes, as well as psychosocial factors, which are known to be associated with the outcomes. Autistic traits were assessed using the Comprehensive Autistic Traits Inventory (CATI), which measures six types of autistic traits rather than a single score. Several aspects of sleep duration (total duration and number of awakenings) and sleep quality were also measured. The next sections describe these features of the research in detail. Data were collected from a large sample of university students. Analyses not only examined the associations between the variables of interest but also adjusted for potential confounders.

### 1.1. Autistic Traits

The broad autism phenotype (BAP) involves individuals who display behavioural characteristics and personality traits that are similar to, but milder than, autistic individuals (i.e., individuals who have been diagnosed with an autism spectrum disorder or ASD) [1]. Family members of autistic individuals often fall under the BAP; however, this also commonly occurs in the general population. Since BAP is not a clinical diagnosis recognised in the diagnostic classification systems, it is difficult to identify, report and provide interventions. By looking at the definition and diagnostic criteria of ASD, we can approach closer to understanding what BAP entails. Autism spectrum disorder is a neurodevelopmental disorder causing difficulties in social interactions and communication, often represented through social camouflage, cognitive rigidity, repetitive behaviours, and sensory sensitivity [2]. These impairments are also linked with difficulties in developing emotional and motor skills and learning language and speech. The severity of these traits determines whether the individual is diagnosed with ASD. When the individual’s symptoms are not severe enough for an ASD diagnosis, it can be said that they display autistic traits, which may impact various aspects of their day-to-day life. Gender is one factor that often impacts the diagnosis of autism. While past research on autism suggests that autistic females tend to engage in social camouflaging more than autistic males, which is one example of gender differences in autistic experiences, reviewed research on individuals with autistic traits is inconclusive [2]. Some studies report no differences based on gender, whereas others show a difference, some that align with the experiences of autistic individuals and others that report the opposite.

While BAP can be difficult to identify, many researchers have used interviews, questionnaires, and neuroimaging to determine the characteristics that define BAP. The Comprehensive Autistic Traits Inventory (CATI) is one such questionnaire that focuses on the six autistic traits mentioned above, namely social interactions (confidence and comfortability with social interactions), communication (understanding verbal and non-verbal cues), social camouflage (copying or ‘mirroring’ the behaviour of others during social interactions), cognitive rigidity (need for routine and order, often representing a pattern), repetitive behaviours (fiddling repetitively for emotional regulation), and sensory sensitivity (sensitivity to sensory cues, i.e., light, sound, smell, touch and taste) [3]. CATI was developed as none of the existing measures considered all the traits associated with ASD as traits such as sensory sensitivity were only recently added to the Diagnostic and Statistical Manual of Mental Disorders fifth edition, revised (DSM-5-TR) [4]. The total score of CATI can be used similarly to Autism Spectrum Quotient-10 (AQ-10), which many studies have used to indicate the presence of autistic traits. However, the advantage of the CATI is that each subscale quantifies the different elements that embody the experiences of individuals with ASD and under the BAP. CATI can help individuals with autistic traits identify what areas they struggle with, which can then be correlated with different aspects of their well-being that may be impacted because of it.

### 1.2. Conceptualising Well-Being

While it is difficult to fully define well-being, as it is a complex and multi-faceted social construct, the well-being process (WBP) model, based on the Demands–Resources–Individual–Effects (DRIVE) model of well-being [5], considers factors in everyday life, such as stress, and categorises them into predictors, mediators, and outcomes. These take the form of general characteristics, individual characteristics, appraisals, and outcomes, all of which can be positive and negative. While the DRIVE model was mainly used to explain well-being in a workplace, the WBP model explains well-being in the general population. It states that many general (social support and demands) and individual (positive personality and negative coping) characteristics when mediated by common appraisals (life satisfaction and perceived stress), can predict positive (happiness) and negative (anxiety and depression) outcomes of well-being. In short, social support, demands, positive personality, and negative coping are predictors of well-being; life satisfaction and perceived stress are mediators; and happiness, anxiety, and depression are the well-being outcomes [2].

The factors considered in the WBP model are vast and difficult to quantify. However, the well-being process questionnaire (WBPQ) breaks down each aspect of the WBP model into quantifiable variables. This questionnaire has two variations—one for workers and the other for students—considering individuals’ struggles regarding their groups. Hence, the students’ version of WBPQ focuses on the impact of academic life and workload on the student’s well-being, such as feelings of burnout [6]. While the WBPQ omits many factors that an individual’s well-being encompasses, such as a sense of purpose and daily functioning, it provides an opportunity to focus on academic-specific factors, such as workload and time management, and explore how these factors may impact an individual’s well-being outcomes such as their perceived stress, and their feelings of anxiety and depression.

University students, neurotypical or neurodivergent, often experience high levels of stress as they need to adapt to new environments and take on responsibilities that they were not expected to take on previously. In addition, many university students find it challenging to maintain a work–life balance at first, which can cause them much academic stress. When combined with neurodivergence, time management can prove difficult due to impaired cognitive flexibility and a need for routine. Many autistic students find university to be an overwhelming sensory experience, which can cause a decline in academic performance, leading to feelings of anxiety and depression. Additionally, social interactions become challenging as autistic students feel a greater need to mask, making socialising exhausting and less rewarding. This can cause an increase in levels of loneliness, leading to more feelings of depression and anxiety [2].

Undoubtedly, mental health plays an important role in an individual’s well-being. Hence, it is important to understand the role that comorbid disorders such as depression, anxiety, and ADHD can have in the well-being of individuals with autistic traits. As discussed, depression and anxiety are some of the primary well-being outcomes for autistic university students. When considering the co-occurrence of ADHD with ASD, these disorders have some overlap in symptoms, especially in the perception and experiences of individuals who exhibit these disorders. Like autistic people, those with ADHD tend to have social difficulties and language impairments that can cause distress and loneliness [7]. This is consistent in university students, where, like autistic students, students with ADHD find a university to be an ‘overwhelming sensory experience’, making it important to consider ADHD as a comorbid disorder when studying autistic traits [2]. Some of these factors have been considered by the Strengths and Difficulties Questionnaire, such as hyperactivity and problems in peer relationships [8].

### 1.3. Sleep Quality

One factor that tends to impact well-being in the general population, but especially in autistic people and people with ADHD, is sleep quality, which is defined as an individual’s satisfaction with their overall sleep experience, including sleep efficiency, sleep latency, sleep duration and wake-after-sleep onset [9]. Many factors, such as age, stress, and mental health, can determine sleep quality. It can lead to exhaustion and fatigue, causing a strain on different aspects of an individual’s life, such as emotional regulation, social relationships, and academic performance. In autistic people and people with ADHD, a lack of sleep can cause a decline in the ability to stay attentive and control emotions, which can trigger impulsive behaviours [10,11].

### 1.4. Associations Between the Variables

Limited research has explored the associations between sleep quality, autistic traits, and well-being in university students. However, reviewing associations between some variables can help produce hypotheses for the current study. More specifically, these studies provide some context for the variables of interest in this study [2]. When considering the correlation between sleep and well-being, specifically the factors covered in the WBPQ [12], researchers explored how daytime sleepiness may be associated with academic satisfaction in university students. There was a significant correlation between subjective depression and tiredness and concentration, sleep quality and daytime sleepiness, and anxiety was significantly correlated with tiredness and concentration and sleep quality, with no significant relationship between sleep duration and well-being outcomes. Hence, daytime sleepiness and sleep quality are important variables to consider when understanding well-being. Furthermore, findings from this study also suggest that tiredness and concentration were significantly correlated with overall academic attainment, implying the importance of studying sleep hygiene of university students. The findings on the role of sleep quality in the well-being of autistic individuals are inconclusive, as autistic individuals have varying sleep experiences [2], highlighting the importance of further examining its role.

To add to these findings, few studies have explored the relationship between autistic traits and well-being in secondary school and university students to highlight the aspects that students struggle with in their day-to-day lives with the presence of autistic traits. One explored the correlation between autistic traits and well-being in secondary school students, utilising the WBPQ and Strengths and Difficulties Questionnaire (SDQ) to quantify well-being and the AQ-10 to quantify autistic traits [8]. Autistic traits were significantly correlated with SDQ items such as emotional problems, conduct problems, hyperactivity, and prosocial behaviour, but there was no significant correlation with well-being outcomes. While this finding provides insight into the influence of autistic traits on being social, paying attention, sitting still in one spot, listening to instructions, and emotional regulation, it is important to consider the impact of age on this relationship.

When considering the correlation between autistic traits and well-being in university students, results were similar to the aforementioned study. Conduct problems, hyperactivity, emotional problems, prosocial behaviour, physical health, peer problems, psychological capital, positive coping, and social support were significantly correlated with autistic traits [13]. Autistic traits were also significantly associated with anxiety and depression, which emphasises the impact autistic traits can have on mental health. The findings of both studies imply that students (either in school or university) often struggle with being in school or university settings when they experience autistic traits. However, it is still unclear if factors specific to students, such as academic workload and time management, can significantly impact the well-being of all students, regardless of neurodivergence, but also how these factors may impact students under the BAP differently from neurotypical students.

### 1.5. Aims

This study aims to understand the link between sleep quality, mental well-being, and autistic traits in university students. The research also aims to understand the effect of individual needs on mental well-being, provide research evidence for the impacts of sleep on well-being in an adult population and emphasise the impacts of autistic traits on mental well-being and how differences in sleep patterns compromise it. Finally, this research aims to extend findings to adults with an ASD diagnosis and their carers by exploring its practical implications and demonstrating the importance of good quality and quantity of sleep regardless of the presence of autistic traits.

## 2. Methods

The research was carried out with the informed consent of the participants and the approval of the Ethics Committee, School of Psychology, Cardiff University. (EC20.03.10.5987R2A3).

Past research utilised the AQ-10 to quantify autistic traits. However, this has its limitations. Hence, the present research used the CATI due to the depth it provides in understanding autistic traits. Additionally, the study focuses on the well-being of university students to highlight the impact of factors specific to students, as quantified by the WBPQ, on well-being and its association with autistic traits. A questionnaire was collated, including four pre-existing measures explained below.

### 2.1. Participants

Demographic information has been summarised in Table 1. Three hundred and eight participants (28 males, 279 females, 1 unreported) were recruited via convenience sampling for the study through the Cardiff University EMS system, which restricts participation to psychology students. Four of these participants only provided demographic information. They did not provide or offer any responses for the remainder of the survey, and 43 participants had some missing data for one to three questions. Three hundred and four participants reported being between 18 and 24 years old, and four were between 25 and 44. One hundred fifty students reported being in their first year of study, 150 in their second year, 1 in their third year, one in their fourth year, one doing their master’s study, and five unreported. The survey was cross-sectional, and all the participants completed the same questionnaire.

### 2.2. Measures

***Smith Short Form Sleep Questionnaire.*** This scale (Appendix A) comprised six items regarding the average quality and quantity of sleep the participants undertake. Two of these questions required participants to input the number of hours of sleep they take (less than four hours, five to eight hours, or more than eight hours) and the number of times they wake up during the night (less than two times, two to five times or more than five times). Three questions were regarding how often participants have difficulty sleeping, waking up before intended, and daytime sleepiness. The participants were asked to rate their agreement with the statement on a 10-point scale (1 = Never and 10 = Very frequently). Finally, one question about sleep quality was rated on a 10-point scale (1 = Extremely Poor and 10 = Very Good). Daytime sleepiness was recorded as part of the SFWBPQ. Cronbach’s alpha score for sleep quality was 0.71, indicating good internal consistency.

***Short-Form Student Well-being Process Questionnaire.*** This scale (Appendix B) consisted of 21 statements. Three of these statements asked about the participants’ demographics: year of study, gender, and age. The remaining 18 questions were each regarding positive well-being, negative well-being, social support, positive coping, negative coping, resilience, psychological capital, flourishing, student stressors, academic stress, work–life balance, flow, rumination, physical health, stress, daytime sleepiness, life satisfaction, feeling anxious and feeling depressed. Each statement was rated on a 10-point scale (1 = Not at all and 10 = Very much so). Scores range from 18 to 180. Cronbach alpha scores for well-being predictors ranged from 0.70 to 0.73, and well-being outcomes ranged from 0.79 to 0.88, indicating good internal consistency.

***Short-Form Strengths and Difficulties Questionnaire.*** This questionnaire (Appendix C) was a short version [8] of the Strengths and Difficulties Questionnaire, created in 1994 [14] as a parent and teacher report measure for children and adolescents. The questionnaire concerned the children’s day-to-day emotional well-being, emotional symptoms, conduct problems, hyperactivity, peer relationship problems, and prosocial behaviours. Initially, the questionnaire consisted of 25 questions, with each “difficulty” comprising five questions. This was narrowed to five questions, one representing each “difficulty”. Participants rated their agreement on a 10-point scale (1 = Not at all, and 10 = Very much so). Scores range from 5 to 50.

***Comprehensive Autistic Traits Inventory.*** This scale (Appendix D) was developed based on autistic traits displayed by autistic people, such as social interaction, communication, social camouflage, cognitive rigidity, repetitive behaviour, and sensory sensitivity [3]. The scale consists of 42 items, with each trait consisting of seven items. Participants rated their agreement with each statement on a five-point scale (1 = Definitely disagree and 5 = Agree) with five reverse-ranked items (items 8, 15, 19, 23 and 28). Cronbach’s alpha scores for each subscale ranged from 0.81 to 0.94, indicating good internal consistency. Scores range from 42 to 210, and higher scores indicate a broad representation of autistic-like traits.

### 2.3. Procedure

The questionnaire, which is made up of scales in Appendix A, Appendix B, Appendix C and Appendix D, was created on Qualtrics using the above measures. The survey was then posted onto the Cardiff University EMS system, where students were awarded two credits to participate in the study. The survey took about 20–30 min to complete. Data collection was completed over two months. Consent was obtained at the start of the questionnaire, and the participants were given a debriefing note at the end.

## 3. Results

Analysis was conducted using IBM SPSS Statistics software, version 29.0.1.1. First, descriptive statistics for each item of the WBPQ (Appendix E), SDQ (Appendix F), Sleep Questionnaire (Appendix G), and the individual CATI subscales (Appendix H) were conducted, presenting the mean and standard deviations. The three components of the WBPQ, predictors, mediators, and outcomes, were kept separate during analyses. The predictors were the factors of social support, positive coping, negative coping, resilience, psychological capital, student stressors, academic stress, work–life balance, flow, and rumination. The mediators (initially treated as outcomes) were stress and life satisfaction. Finally, positive well-being, negative well-being, flourishing, physical health, anxiety, and depression were the outcomes. Using this, correlations between predictors and mediators and between predictors and outcomes were established, and the significant associations between sleep and outcomes are presented in Table 2.

The data were then subjected to ANOVA and MANCOVA. Analyses of Variance (ANOVA) were then conducted to assess the statistical effect of the CATI subscales, sleep quality, and their interaction with the WBPQ and SDQ items. A multivariate covariance (MANCOVA) analysis assessed the overall significant effect of CATI subscales, well-being predictors, and sleep questions as covariates with the SDQ items and well-being variables as dependent variables. Finally, analysis was also conducted with CATI subscales as covariates and well-being predictors as the dependent variables.

Significant correlations between the CATI scores and the outcomes are shown in Table 3.

### 3.1. ANOVA, Including All Three Variables

Before analysis, sleep quality and CATI subscale scores were divided into ‘high’ and ‘low’ groups on SPSS based on overall percentile scores.

The ANOVA showed that better sleep quality was associated with fewer conduct problems (*F* = 4.01, *p* < 0.05), greater positive well-being (*F* = 10.53, *p* < 0.005), greater flourishing (*F* = 4.00, *p* < 0.05), better physical health (*F* = 6.57 *p* < 0.05), greater life satisfaction (*F* = 11.71, *p* < 0.001) and lower levels of depression (*F* = 5.19, *p* < 0.05).

Analysis of the CATI subscales showed that greater social camouflage (CATI_CAM) was associated with lower flourishing (*F* = 9.47, *p* < 0.005), higher levels of depression (*F* = 5.01, *p* < 0.05), greater hyperactivity (*F* = 8.97, *p* < 0.005), more conduct problems (*F* = 4.55, *p* < 0.05) and more emotional problems (*F* = 19.81, *p* < 0.001). Better communication (CATI_COM) was associated with less hyperactivity (*F* = 8.43, *p* < 0.005), fewer conduct problems (*F* = 15.32, *p* < 0.001), and lower prosocial behaviour (*F* = 5.64, *p* < 0.05).

More repetitive behaviours (CATI_REP) were associated with greater perceived stress (*F* = 4.99, *p* < 0.05), lower life satisfaction (*F* = 4.41, *p* < 0.05), greater levels of anxiety (*F* = 11.06, *p* < 0.005) and depression (*F* = 4.727, *p* < 0.05), more significant hyperactivity (*F* = 81.83, *p* < 0.001), more conduct problems (*F* = 9.08, *p* < 0.005) and more emotional problems (*F* = 21.82, *p* < 0.001). Greater cognitive rigidity (CATI_RIG) was associated with greater negative well-being (*F* = 5.69, *p* < 0.05), more significant hyperactivity (*F* = 12.32, *p* < 0.001), more conduct problems (*F* = 7.44, *p* < 0.01) and more emotional problems (*F* = 4.08, *p* < 0.05).

Greater sensory sensitivity (CATI_SEN) was associated with greater negative well-being (*F* = 6.43, *p* < 0.05), higher levels of depression (*F* = 7.47, *p* < 0.01), greater hyperactivity (*F* = 36.44, *p* < 0.001), more conduct problems (*F* = 12.79, *p* < 0.001), more emotional problems (*F* = 5.23, *p* < 0.05), fewer peer problems (*F* = 7.17, *p* < 0.01) and lower prosocial behaviour (*F* = 5.45, *p* < 0.05). Fewer social interactions (CATI_SOC) were associated with less hyperactivity (*F* = 11.15, *p* < 0.001), fewer emotional problems (*F* = 8.80, *p* < 0.005), and more peer problems (*F* = 9.61, *p* < 0.005).

The interaction between sleep quality and the CATI subscales had non-significant effects on the WBPQ and SDQ items: ‘CATI_CAM’ (Wilks’ Λ = 0.94, *p* = 0.26), ‘CATI_COM’ (Wilks’ Λ = 0.97, *p* = 0.76), ‘CATI_REP’ (Wilks’ Λ = 0.94, *p* = 0.20), ‘CAT_RIG’ (Wilks’ Λ = 0.95, *p* = 0.47), ‘CATI_SEN’ = (Wilks’ Λ = 0.95, *p* = 0.42) and ‘CATI_SOC’ (Wilks’ Λ = 0.97, *p* = 0.76).

### 3.2. MANCOVA, Including the Well-Being Predictors

#### 3.2.1. CATI Subscales as Covariates

Table 4 summarises the MANCOVA results for the CATI subscales as covariates and SDQ and WBPQ items as dependent variables. When the overall significance of the CATI subscales was assessed, ‘CATI_SOC’ (Wilks’ Λ = 0.95, *p* < 0.05), ‘CATI_CAM’ (Wilks’ Λ = 0.95, *p* < 0.05), and ‘CATI_REP’ (Wilks’ Λ = 0.86, *p* < 0.001) had a statistically significant effect for the SDQ items. More specifically, more social interactions were associated with more emotional problems (β = 0.720, F = 6.15, *p* < 0.05) and fewer peer problems (β = −0.590, F = 7.74, *p* < 0.01). Greater social camouflaging was associated with more emotional problems (β = 0.768, F = 5.81, *p* < 0.05) and fewer peer problems (β = −0.528, F = 5.15, *p* < 0.05). Finally, more repetitive behaviours were associated with greater hyperactivity (β = 1.856, F = 39.83, *p* < 0.001), more emotional problems (β = 0.699, F = 4.84, *p* < 0.05), and reduced prosocial behaviour (β = −0.501, F = 5.60, *p* < 0.05).

‘CATI_RIG’ (Wilks’ Λ = 0.92, *p* < 0.01) and ‘CATI_REP’ (Wilks’ Λ = 0.93, *p* < 0.01) had a statistically significant effect on the well-being outcomes. Greater cognitive rigidity was associated with higher negative well-being (β = 0.690, F = 6.08, *p* < 0.05) and greater perceived stress (β = 0.549, F = 5.232, *p* < 0.05). More repetitive behaviours were associated with worse physical health (β = −0.521, F = 4.14, *p* < 0.05) and greater levels of anxiety (β = 0.752, F = 6.41, *p* < 0.05).

#### 3.2.2. Well-Being Predictors as Covariates

The significant effects of the well-being predictors are shown in Table 5.

‘Social Support’ (Wilks’ Λ = 0.94, *p* < 0.005), ‘Negative Coping’ (Wilks’ Λ = 0.91, *p* < 0.001), ‘Resilience’ (Wilks’ Λ = 0.93, *p* < 0.005), ‘Psychological Capital’ (Wilks’ Λ = 0.93, *p* < 0.001) and ‘Student stressors’ (Wilks’ Λ = 0.89, *p* < 0.001) had a statistically significant effect on the SDQ items. More specifically, greater social support was associated with fewer peer problems (*β* = 0.226, *F* = 12.98, *p* < 0.001). Greater use of negative coping was associated with greater hyperactivity (*β* = 0.211, *F* = 8.99, *p* < 0.005), more emotional problems (*β* = 0.232, *F* = 15.54, *p* < 0.001), and more peer problems (*β* = 0.100, *F* = 4.39, *p* < 0.05). Greater resilience was associated with fewer peer problems (*β* = −0.197, *F* = 12.32, *p* < 0.001) and greater prosocial behaviour (*β* = 0.196, *F* = 13.94, *p* < 0.001). Higher psychological capital was associated with decreased emotional problems (*β* = −0.334, *F* = 14.00, *p* < 0.001). Finally, more student stressors were associated with more emotional problems (*β* = 0.319, *F* = 21.57, *p* < 0.001) and more prosocial problems (*β* = −0.177, *F* = 11.37, *p* < 0.001).

When considering the well-being outcomes, ‘Social Support’ (Wilks’ Λ = 0.86, *p* < 0.001), ‘Negative Coping’ (Wilks’ Λ = 0.90, *p* < 0.001), ‘Resilience’ (Wilks’ Λ = 0.93, *p* < 0.01), ‘Psychological Capacity’ (Wilks’ Λ = 0.77, *p* < 0.001), ‘Student stressors’ (Wilks’ Λ = 0.87, *p* < 0.001), ‘Academic Stress’ (Wilks’ Λ = 0.82, *p* < 0.001), and ‘Flow’ (Wilks’ Λ = 0.83, *p* < 0.001) had overall significant effects. More specifically, greater social support was associated with greater positive well-being (*β* = 0.212, *F* = 10.38, *p* < 0.005), greater flourishing (*β* = 0.181, *F* = 15.69, *p* < 0.001), better physical health (*β* = 0.198, *F* = 9.53, *p* < 0.005), greater life satisfaction (*β* = 0.187, *F* = 15.21 *p* < 0.001) and feeling less depressed (*β* = −0.329, *F* = 20.08, *p* < 0.001). Greater use of negative coping was associated with greater negative well-being (*β* = 0.208, *F* = 13.10, *p* < 0.001), higher levels of perceived stress (*β* = 0.162, *F* = 15.46, *p* < 0.001), and greater levels of anxiety (*β* = 0.204, *F* = 14.59, *p* < 0.001) and depression (*β* = 0.257, *F* = 17.45, *p* < 0.001). Greater resilience was associated with greater negative well-being (*β* = 0.135, *F* = 4.20, *p* < 0.05), better physical health (*β* = 0.151, *F* = 6.01, *p* < 0.05), and feeling greater levels of depression (*β* = 0.159, *F* = 5.10, *p* < 0.05).

High psychological capital was associated with greater positive well-being (*β* = 0.367, *F* = 20.27, *p* < 0.001), lower negative well-being (*β* = −0.282, *F* = 10.98, *p* < 0.005), greater flourishing (*β* = 0.436, *F* = 59.15, *p* < 0.001), better physical health (*β* = 0.174, *F* = 4.76, *p* < 0.05), greater life satisfaction (*β* = 0.304, *F* = 26.20, *p* < 0.001), lower perceived stress (*β* = −0.171, *F* = 7.92, *p* < 0.01), and lower levels of anxiety (*β* = −0.294, *F* = 13.85, *p* < 0.001) and depression (*β* = −0.373, *F* = 16.80, *p* < 0.001). Furthermore, more student stressors were associated with greater negative well-being (*β* = 0.220, *F* = 10.99, *p* < 0.005), lower flourishing (*β* = −0.115, *F* = 6.75, *p* < 0.05), greater perceived stress (*β* = 0.273, *F* = 32.91, *p* < 0.001), and feeling more anxious (*β* = 0.264, *F* = 18.36, *p* < 0.001) and depressed (*β* = 0.249, *F* = 12.33, *p* < 0.001). Greater academic stress was associated with greater negative well-being (*β* = 0.238, *F* = 12.90, *p* < 0.001), lower flourishing (*β* = −0.099, *F* = 5.03, *p* < 0.05), greater perceived stress (*β* = 0.318, *F* = 44.73, *p* < 0.001) and feeling more anxious (*β* = 0.190, *F* = 9.54, *p* < 0.005). Finally, greater flow was associated with greater flourishing (*β* = 0.265, *F* = 42.94, *p* < 0.001) and greater life satisfaction (*β* = 0.185, *F* = 19.20, *p* < 0.001).

#### 3.2.3. Sleep Questions

Table 6 summarises the MANCOVA results for the sleep questions as covariates and SDQ and WBPQ items as dependent variables.

Amongst the Sleep Questions, ‘Difficulty Sleeping’ (Wilks’ Λ = 0.95, *p* < 0.01), ‘Sleep Quality’ (Wilks’ Λ = 0.95, *p* < 0.01) and ‘Daytime Sleepiness’ (Wilks’ Λ = 0.83, *p* < 0.001) had an overall statistically significant effect on SDQ items. More specifically, greater difficulty falling asleep was associated with more hyperactivity (*β* = 0.166, *F* = 6.86, *p* < 0.01) and more emotional problems (*β* = 0.167, *F* = 8.00, *p* < 0.01). Better sleep quality was associated with fewer peer problems (*β* = −0.213, *F* = 10.07, *p* < 0.005). Finally, more significant daytime sleepiness was associated with greater hyperactivity (*β* = 0.236, *F* = 18.05, *p* < 0.001) and more emotional problems (*β* = 0.356, *F* = 45.73, *p* < 0.001).

‘Difficulty Sleeping’ (Wilks’ Λ = 0.89, *p* < 0.001), ‘Sleep Quality’ (Wilks’ Λ = 0.80, *p* < 0.001), and ‘Daytime Sleepiness’ (Wilks’ Λ = 0.83, *p* < 0.001) had a statistically overall significant effect on the well-being outcomes. More specifically, greater difficulty with falling asleep was associated with more perceived stress (*β* = 0.132, *F* = 7.72, *p* < 0.01) and more anxiety (*β* = 0.125, *F* = 5.44, *p* < 0.05) and depression (*β* = 0.277, *F* = 20.51, *p* < 0.001). Better sleep quality was associated with greater positive well-being (*β* = 0.359, *F* = 21.96, *p* < 0.001), greater flourishing (*β* = 0.464, *F* = 48.38, *p* < 0.001), better physical health (*β* = 0.204, *F* = 8.52, *p* < 0.005), greater life satisfaction (*β* = 0.399, *F* = 45.08, *p* < 0.001), lower perceived stress (*β* = −0.166, *F* = 6.34, *p* < 0.05), and lower levels of anxiety (*β* = −0.205, *F* = 7.62, *p* < 0.01) and depression (*β* = −0.206, *F* = 7.03, *p* < 0.01). Finally, more daytime sleepiness was associated with greater negative well-being (*β* = 0.316, *F* = 40.62, *p* < 0.001), lower flourishing (*β* = −0.106, *F* = 6.20, *p* < 0.05), more significant perceived stress (*β* = 0.247, *F* = 34.37, *p* < 0.001), and more anxiety (*β* = 0.284, *F* = 35.63, *p* < 0.001) and depression (*β* = 0.242, *F* = 19.95, *p* < 0.001).

#### 3.2.4. Effects of All Covariates in a Single Analysis

The following analyses included all the covariates in analyses of the SDQ and well-being outcomes. When all three groups of covariates, i.e., CATI subscales, well-being predictors and Sleep Questions, were assessed (as summarised in Table 7), ‘Daytime Sleepiness’ (Wilks’ Λ = 0.94, *p* < 0.05), ‘Social Support’ (Wilks’ Λ = 0.94, *p* < 0.05), ‘Negative Coping’ (Wilks’ Λ = 0.95, *p* < 0.05), ‘Resilience’ (Wilks’ Λ = 0.95, *p* < 0.05), ‘Psychological Capital’ (Wilks’ Λ = 0.93, *p* < 0.005), ‘Student Stressors’ (Wilks’ Λ = 0.90, *p* < 0.001) and ‘CATI_REP’ (Wilks’ Λ = 0.87, *p* < 0.001) had an overall statistically significant effect on the SDQ items.

More specifically, greater daytime sleepiness was associated with more emotional problems (*β* = 0.179, *F* = 13.11, *p* < 0.001). More social support was associated with fewer peer problems (*β* = −0.223, *F* = 12.03, *p* < 0.001). Greater use of negative coping was associated with more emotional problems (*β* = 0.151, *F* = 6.37, *p* < 0.05) and more peer problems (*β* = 0.115, *F* = 4.88, *p* < 0.05). Better resilience was associated with fewer peer problems (*β* = −0.174, *F* = 7.98, *p* < 0.01) and more prosocial behaviour (*β* = 0.160, *F* = 7.72, *p* < 0.01). Greater psychological capital was associated with fewer emotional problems (*β* = −0.338, *F* = 14.19, *p* < 0.001). More student stressors were associated with greater emotional problems (*β* = 0.310, *F* = 20.13, *p* < 0.001) and reduced prosocial behaviour (*β* = −0.141, *F* = 6.30, *p* < 0.05). Finally, more repetitive behaviours were associated with greater hyperactivity (*β* = 1.762, *F* =33.84, *p* < 0.001).

Considering the well-being outcomes (summarised in Table 8), ‘Difficulty Sleeping’ (Wilks’ Λ = 0.91, *p* < 0.005), ‘Sleep Quality’ (Wilks’ Λ = 0.92, *p* < 0.05), ‘Daytime Sleepiness’ (Wilks’ Λ = 0.87, *p* < 0.001), ‘Social Support’ (Wilks’ Λ = 0.88, *p* < 0.001), ‘Negative Coping’ (Wilks’ Λ = 0.91, *p* < 0.005), ‘Resilience’ (Wilks’ Λ = 0.93, *p* < 0.05), ‘Psychological Capital’ (Wilks’ Λ = 0.80, *p* < 0.001), ‘Student Stressors’ (Wilks’ Λ = 0.87, *p* < 0.001) Academic Stress’ (Wilks’ Λ = 0.86, *p* < 0.001), ‘Flow’ (Wilks’ Λ = 0.83, *p* < 0.001), ‘Rumination’ (Wilks’ Λ = 0.93, *p* < 0.05) and ‘CATI_RIG’ (Wilks’ Λ = 0.93, *p* < 0.05) had overall statistically significant effects.

More specifically, greater difficulty with falling asleep was associated with more depression (*β* = 0.203, *F* = 13.81, *p* < 0.001). Better sleep quality was associated with greater flourishing (*β* = 0.152, *F* = 8.58, *p* < 0.005) and greater life satisfaction (*β* = 0.154, *F* = 7.77, *p* < 0.01). Greater daytime sleepiness was associated with greater negative well-being (*β* = 0.211, *F* = 18.44, *p* < 0.001), more perceived stress (*β* = 0.131, *F* = 13.32, *p* < 0.001), and feeling more anxious (*β* = 0.171, *F* = 14.06, *p* < 0.001) and depressed (*β* = 0.130, *F* = 6.32, *p* < 0.05). Greater social support was associated with greater positive well-being (*β* = 0.214, *F* = 9.04, *p* < 0.005), greater flourishing (*β* = 0.201, *F* = 16.41, *p* < 0.001), better physical health (*β* = 0.177, *F* = 5.73, *p* < 0.05), greater life satisfaction (*β* = 0.170, *F* = 10.39, *p* < 0.005) and lower levels of depression (*β* = −0.260, *F* = 11.24, *p* < 0.001).

Greater use of negative coping was associated with greater negative well-being (*β* = 0.141, *F* = 5.68, *p* < 0.05), lower flourishing (*β* = −0.112, *F* = 7.91, *p* < 0.01), more perceived stress (*β* = 0.137, *F* = 10.02, *p* < 0.005), and more anxiety (*β* = 0.179, *F* = 10.60, *p* < 0.005) and depression (*β* = 0.215, *F* = 11.90, *p* < 0.001). Greater resilience was associated with better physical health (*β* = 0.195, *F* = 7.90, *p* < 0.01) and greater life satisfaction (*β* = 0.119, *F* = 5.76, *p* < 0.05). High psychological capital was associated with greater positive well-being (*β* = 0.304, *F* = 13.07, *p* < 0.001), lower negative well-being (*β* = −0.211, *F* = 5.85, *p* < 0.05), greater flourishing (*β* = 0.388, *F* = 43.98, *p* < 0.001), greater life satisfaction (*β* = 0.283, *F* = 20.67, *p* < 0.001), and lower levels of anxiety (*β* = −0.236, *F* = 8.47, *p* < 0.005) and depression (*β* = −0.318, *F* = 12.01, *p* < 0.001).

More student stressors were associated with greater negative well-being (*β* = 0.215, *F* = 9.93, *p* < 0.005), lower flourishing (*β* = −0.136, *F* = 8.84, *p* < 0.005), more perceived stress (*β* = 0.265, *F* = 28.30, *p* < 0.001), and more anxiety (*β* = 0.253, *F* = 15.94, *p* < 0.001) and depression (*β* = 0.217, *F* = 9.10, *p* < 0.005). Greater academic stress was associated with greater negative well-being (*β* = 0.173, *F* = 6.48, *p* < 0.05), lower flourishing (*β* = −0.094, *F* = 4.27, *p* < 0.05), greater perceived stress (*β* = 0.248, *F* = 24.80, *p* < 0.001) and greater life satisfaction (*β* = 0.097, *F* = 3.98, *p* < 0.05). Greater flow was associated with greater flourishing (*β* = 0.241, *F* = 34.49, *p* < 0.001) and better life satisfaction (*β* = 0.165, *F* = 14.30, *p* < 0.001). More positive rumination was associated with positive well-being (*β* = 0.144, *F* = 8.63, *p* < 0.005). Finally, greater cognitive rigidity was associated with greater negative well-being (*β* = 0.672, *F* = 7.71, *p* < 0.01).

### 3.3. CATI Subscales and Well-Being Predictors

The overall score for each CATI subscale was used for this MANCOVA analysis. Table 9 summarises the results for the CATI subscales as covariates and WBPQ predictors as dependent variables.

In this analysis, ‘CATI_COM’ (Wilks’ Λ = 0.91, *p* < 0.01), ‘CATI_CAM’ (Wilks’ Λ = 0.82, *p* < 0.001), and ‘CATI_RIG’ (Wilks’ Λ = 0.91, *p* < 0.01) had an overall statistically significant effect on the WBPQ predictors. More specifically, greater problems with communication were associated with lower resilience (*β* = −0.102, *F* = 6.67, *p* < 0.05) and worse work–life balance (*β* = −0.097, *F* = 4.71, *p* < 0.05). Greater social camouflaging was associated with less social support (*β* = −1.105, *F* = 29.70, *p* < 0.001), less use of positive coping (*β* = −0.116, *F* = 25.25, *p* < 0.001), greater use of negative coping (*β* = 0.053, *F* = 4.18, *p* < 0.05) lower resilience (*β* = −0.071, *F* = 9.10, *p* < 0.005), lower psychological capacity (*β* = −0.060, *F* = 7.43, *p* < 0.01) and greater rumination (*β* = 0.055, *F* = 4.28, *p* < 0.05). Finally, greater cognitive rigidity was associated with more social support (*β* = 0.077, *F* = 11.06, *p* < 0.005), greater use of positive coping (*β* = 0.051, *F* = 4.31, *p* < 0.05), and better flow (*β* = 0.058, *F* = 5.86, *p* < 0.05).

## 4. Discussion

The present study succeeded in replicating some past findings on the topic and providing some novel results to add to past research. ANOVA results on the associations between autistic traits and well-being often aligned with findings from past research. The MANCOVA results are novel and provide insight into how sleep quality and daytime sleepiness interact with different autistic traits to influence overall well-being. Like past research, the findings of the present study aid in understanding the correlation between sleep quality, autistic traits, and well-being. This helps in determining whether these findings support or refute conclusions drawn in past research. It should be noted that most research studies focused on the overall score on scales that quantify autistic traits, whereas the present study focuses on individual autistic traits, presenting novel findings on the correlation between each autistic trait, sleep quality, and well-being. The findings of this study also help review the reliability and consistency of the WBPQ, SDQ, and CATI.

### 4.1. Interpretation of Results

This study examined the relationship between sleep quality, autistic traits, and well-being by examining associations between each item in the WBPQ, SDQ, CATI, and Sleep Questionnaires. First, the univariate correlations in Table 1 and Table 2 show the correlations between autistic traits, sleep quality, well-being predictors, and SDQ and well-being outcomes. This highlighted that better sleep quality was associated with more positive well-being, such as feeling happy, in good spirits, feeling good about relationships, etc., greater flourishing, thriving, being successful, feeling that life is going well, and having a sense of belonging, better physical health, feeling less depressed, greater life satisfaction and having fewer instances of anger, conduct problems and dishonesty.

Amongst the autistic traits, five of six autistic traits (excluding social interactions) were associated with feeling more hyperactive, fidgety, and impulsive and having more significant instances of anger, conduct problems, and dishonesty. All autistic traits except communication were associated with more significant emotional problems, such as worrying a lot, feeling unhappy or downhearted, and being nervous in new situations. Cognitive rigidity and sensory sensitivity were associated with greater negative well-being, such as feeling stressed and emotionally drained. Sensory sensitivity and social interactions were associated with more peer problems, including not having good friends and feeling alone. Difficulty with communication and sensory sensitivity were associated with difficulty in considering other people’s feelings and being kind and helpful. Social camouflaging, repetitive behaviours, and sensory sensitivity were associated with feeling more depressed. Repetitive behaviours were also associated with more perceived stress and feeling more anxious. Although past research focused primarily on autistic individuals, the presence of autistic traits with no diagnosis appears to show similar results.

ANOVA analyses helped understand the overall variance due to sleep and autistic traits in overall well-being, which comprised the WBPQ and SDQ items. The interaction between autistic traits and sleep quality was non-significant; hence, the main effects were considered. When the main effects of the variables were considered, repetitive behaviours had the most significant impact on SDQ outcomes, and sleep quality had a less significant effect on well-being outcomes and SDQ items than all autistic traits. While sleep, well-being predictors, and autistic traits caused a significant variance in SDQ items and well-being outcomes individually, the variance caused by sleep and well-being predictors on SDQ items and well-being outcomes did not vary with the presence of autistic traits. This implies that better sleep quality can predict better overall well-being, regardless of the presence of autistic traits, even though the presence of autistic traits significantly impacts well-being. It can be concluded that autistic traits were primarily associated with SDQ outcomes, whereas well-being predictors predicted well-being outcomes. Sleep quality has some effects on both SDQ and well-being outcomes. These findings are consistent with another study that used the SDQ alongside AQ instead of CATI, highlighting the impact autistic traits have on emotional well-being and resulting behaviours [13].

Considering that the SDQ measures the emotional well-being of individuals based on psychosocial factors, social camouflaging has been found to significantly impact emotional well-being due to the need for social acceptance, maintaining self-esteem and identity, etc., as reported in mixed methods systematic review [15]. Autistic individuals tend to camouflage when they feel the need to conform to social norms and appear neurotypical, usually to avoid instances of bullying and victimisation. This leads to them feeling overlooked, under-supported, and burnt out as their needs are unmet due to prioritising social acceptance. Camouflaging is also often a symbol of self-acceptance and -advocacy for autistic individuals. However, it can have the opposite effect and cause low self-esteem and confusion [15].

MANCOVA analyses were carried out for two main reasons. First, including all the variables from one category (e.g., all the CATI scales) enables one to adjust for the effects of the other scales when considering any specific scale. Secondly, the CATI scores, sleep scores, and well-being predictors adjust for the different categories and the items in the same category of predictors. The MANCOVA with all the CATI scales as predictors showed that different CATI scores had selective effects on the SDQ outcomes. In contrast, only the cognitive rigidity and repetitive behaviour scores were significantly associated with the WPQ outcomes, with cognitive rigidity being more significant. The results were different from the previous associations between the CATI subscales and well-being outcomes. In a mediation analysis, it was found that perfectionism negatively predicted cognitive flexibility, i.e., a greater need for perfectionism predicted low cognitive flexibility. This relationship was mediated by self-compassion and psychological well-being, where people with greater self-compassion and better psychological well-being (which consists of meaning and purpose, engagement, quality of peer relationships, self-acceptance, competency, optimism, and perceived respect) often had more cognitive flexibility [16]. This aligns with the findings of the present study, as cognitive rigidity was associated with negative well-being and perceived stress, suggesting that this may be due to the quality of the individual’s psychological well-being.

Amongst the well-being predictors, student stressors had the most significant effect on SDQ items, which implies that stress factors specific to students, such as academic dissatisfaction and time pressure, impact their emotional well-being. This aligns with the previously mentioned findings that university students report feeling stressed and burnt out due to their workload and the need for adaptation and socialisation in university [2,6]. Psychological capacity had the most significant effect on well-being outcomes. Psychological capacity or capital involves an individual’s ability to make decisions, understand information, and communicate. Research on university students has reported that better psychological capacity was associated with better psychological well-being due to their use of adaptive coping strategies [17]. Having better psychological well-being impacted the students’ problem-solving capabilities and their seeking of emotional and practical social support [17].

Of the sleep questions, daytime sleepiness had the most significant effect on SDQ items. Researchers investigated how social jetlag, the discordance between the body and the societal clock, correlated with behavioural problems [18]. They found that daytime sleepiness caused by social jetlag was associated with behavioural difficulties, conduct problems, hyperactivity, and peer relationship problems, which aligns with the present study. They suggested a biological and social mechanism to explain this correlation, where social jetlag may impact the transmission of dopamine in the amygdala (primarily responsible for emotional processing) and ventral striatum (responsible for decision-making, emotion formation, and reward-seeking behaviour), which may change reward-seeking behaviour causing behavioural problems. Individuals experience low levels of biological alertness, which can impact their involvement in social activities [18]. This study was conducted on adolescents with stressors similar to university students, such as academic workload. Additionally, sleep quality had the most significant effect on well-being outcomes. In university students, due to their stressors, poor sleep quality is often associated with academic dissatisfaction due to tiredness and difficulty concentrating, which is correlated with higher levels of anxiety [12], which aligns with the findings of the current study.

When all of the covariates were included in a single analysis, there were few significant associations between the SDQ scores and the sleep and CATI variations. In contrast, the WPQ predictors showed associations with emotional problems, peer problems, and prosocial behaviour. This profile was replicated with the well-being outcomes, with the well-being predictors showing the usual profile of associations and the sleep and CATI scores having fewer significant effects. This absence of significant effects on the sleep and CATI variables may be due to their association with well-being predictors. This suggests future mediation/moderation analysis.

It is important to consider why these are the only significant associations that involve aspects of the sleep questionnaire. Firstly, it should be noted that the aspects of sleep that had significant correlations with well-being predictors and autistic traits and that cause a significant variance in well-being outcomes and SDQ items are daytime sleepiness and difficulty falling asleep, which are easier to quantify and report than overall sleep quality and how many times participants wake up during the night. In addition, since several hours of sleep had non-significant results, it is not easy to understand if this impacts the other aspects of the sleep questionnaire. For example, researchers reported that people who slept six to eight hours a night had a higher brain volume than those who did not. In addition to this, too much or too little quantity of sleep was correlated with poor memory and an increased risk of dementia [19]. The researchers also reported that seven hours of sleep was associated with the highest cognitive performance, and daytime sleepiness was a negative predictor of executive function [19]. While the population was healthy individuals, ranging from 38 to 73 years of age, in a UK biobank, these findings question the importance of sleep quantity in well-being.

Two factors that could have impacted this in the present study are age and neurodiversity. University students struggle with their sleep due to academic stress and workload. There may be some students who sleep more hours than recommended and others who sleep fewer hours. This may depend on individual differences, such as student coping strategies for academic stress. Another factor that may impact this is problematic smartphone use. Researchers found a significant correlation between compulsive use of social media and poor sleep hygiene, as blue light emissions from smartphones impact melatonin production, which can impact circadian rhythms negatively. This worked both ways, as many individuals used social media to cope with their stress [20,21]. These correlations tended to be significant in students more than in working professionals. This could explain the difference in the quantity of sleep students have.

When considering the impact of neurodivergence, it is essential to consider the construct of the perception of disability, specifically the self-perception and self-concept that autistic people hold. Researchers highlighted in their thematic analysis of themes identified in a focus group discussion that autism is a spectrum experienced differently by every individual based on various factors such as their environment and age [22]. This difference in self-perception amongst the autistic population can impact how each views aspects of their well-being and the factors that might affect it. This can be extended to the current study’s findings, as students with autistic traits may have different sleep experiences and perceived well-being experiences, making it difficult to establish a significant pattern in some of their experiences. This may explain why a non-significant correlation between autistic traits and sleep quality was established. Students with autistic traits may have varying sleep quantity and quality, impacting their perceived well-being differently. Some students, with or without autistic traits, may sleep over eight hours and find that their perceived well-being is poor, whereas others may not sleep enough and report having good overall well-being.

### 4.2. Strengths and Limitations

There are various strengths to this study that are worth noting. Age as a factor in the associations between sleep quality, autistic traits, and well-being can be eliminated, as 304 out of 308 participants were between 18 and 24. This makes the study’s findings focus on a specific population, increasing its generalisability. As they were all university students from the same university and department, their experiences as students were relatively consistent. This demonstrates the significant impact of student stressors and academic stress on some of their well-being outcomes.

While the association between sleep quality and autistic traits is non-significant, this study sheds light on the individual differences between autistic people and people with autistic traits in how they experience and perceive their well-being. It is also important to note that these findings align with some past research in that autistic university students do not see a difference in their sleep quality upon starting university. However, other studies report a significant decline in sleep quality reported by autistic students, which yet again highlights that the experiences and perceptions of autistic individuals may not be consistent.

Another strength of the study is the use of the CATI scale. While the scale can be used to obtain an overall score of the prevalence of autistic traits, using the scale to obtain separate scores of each subscale, or each autistic trait, is important. Through this, the impact of each autistic trait on well-being could be investigated. While there were some overlaps, it is evident that not all traits impact well-being similarly. For example, social camouflaging was associated with peer problems, and communication was related to prosocial problems. Consequently, this highlights that understanding the impact of individual autistic traits on well-being is essential to give some depth to our general understanding of how autistic traits impact well-being. While CATI has good internal consistency, it has not been used in as many studies as other scales, such as Autism Spectrum Quotient. This makes it difficult to test the external validity of this scale and have some level of certainty that there is consistency in the results obtained over different studies, in various scenarios, and with other populations.

Some of the limitations of this study are based on the conceptualising of well-being. Well-being is a vast and complex concept, and while the well-being process model helps in simplifying it by focusing on a handful of aspects of day-to-day life that impact well-being, it can be questioned if it completely encompasses well-being as a concept that often involves many other factors such how one would rate their functioning (ability to get out of bed, brush their teeth, cook) and if they have a sense of meaning and purpose in life which tends to be important in determining an individual’s well-being. While these concepts are difficult to quantify, rating high or low on the WBPQ does not fully show how good one’s well-being is.

On that same note, while using a survey ensures a large sample size, it does not fully encompass the complexity of well-being and ASD, as the type of information gained is limited. Since the scores are self-reported, it is difficult to know the well-being of an individual objectively and if they may have under or overestimated their well-being. Additionally, it is essential to consider the occurrence of demand characteristics. Due to ethical considerations, it is impossible to have any form of deception for surveys as some questions may cause mental harm. This makes it easier for participants, especially psychology students, to guess the study’s aims.

It can also be questioned whether measuring sleep quality cross-sectionally, not longitudinally, would fully represent its role in the association. Overall sleep varies not only based on individual differences such as age, gender, and BMI but also due to external factors such as stress and life demands. In this context, university life can often be inconsistent regarding academic satisfaction, time management, and workload, depending on the time of year. Naturally, when students approach assessment deadlines and exam periods, they may face more issues with their sleep than when these demands are absent. Hence, collecting data on their sleep quality at a particular time may not fully portray the actual quality of their sleep over time.

Finally, demographic information collected, such as gender differences and the students’ disciplines, is another limitation of this study. Since most participants were female (28 males and 279 females), it is difficult to determine if the gender differences impacted the findings. Besides individual differences of autistic people, autism is displayed and expressed differently between males and females. For example, autistic females are more likely to camouflage than autistic males. Additionally, it is more difficult to detect and diagnose autism in females than in males. Furthermore, all the students who participated in the study had a psychology background. While this can indicate some consistency in academic workload and stress experienced by the participants, this also increases the likelihood of demand characteristics, which may impact the validity of the findings. Hence, it is essential to consider the impact that gender and students’ disciplines may have on the present study’s findings.

### 4.3. Future Recommendations

To eliminate some of the limitations of this study, follow-up studies and analyses will be conducted in future studies. First, mediation and moderation analyses will be undertaken to develop a model between autistic traits, sleep quality, and well-being. If any mediation or moderation is observed, it will add to the findings of this study. This could also provide further insight into the role of sleep quality in the association and determine which aspects of the sleep questionnaire tend to have a significant impact. Future studies will also employ a more hands-on approach and test the association (or causation) between these variables by working with some secondary school students. These studies can help understand how sleep quality and the presence of autistic traits vary between age groups, i.e., whether there is a pattern between the two or whether the results are incomparable. This can also help test the validity of present findings by testing groups that have less knowledge about the purpose of the study than psychology students. This will also help to test the external validity of the CATI scale and investigate the consistency of the results produced by this scale. It is also important to consider how sleep quality can vary over time to understand better its role, which will also be implemented. Students will report their sleep quality over two months to better understand the role of sleep quality. Finally, future studies will also be using the ADHDQ and PTSD checklist for DSM-5 to see the impact of past traumatic experiences and ADHD symptoms on autistic traits, sleep quality, and well-being.

### 4.4. Conclusions

The study met the established aim of understanding the correlation between autistic traits, well-being, and sleep quality at university. However, the role of sleep in the well-being of students with autistic traits is still inconclusive. The findings of this study were aligned with past research where autistic traits tend to influence well-being, as does sleep quality. However, there is still the question of causation, and not enough was said about the role of comorbid disorders, such as ADHD and PTSD, in the well-being of autistic people and people with autistic traits, as there is some evidence to suggest that they play a significant role. Future studies aim to consider some of these aspects to better understand the association between sleep quality, autistic traits, and well-being.

## Figures and Tables

**Table 1:** Demographics.

		Frequency	Percent
Gender	Male	28	9.1
	Female	279	90.6
	Missing	1	0.3
	**Range**	**Mean**	**SD**
Age	26	21.18	1.588

**Table 2:** Significant associations between sleep questions and SDQ and WBPQ items.

	Predictors	Outcomes	r	*p*-Value
**SDQ**	Sleep Quality	Conduct problems	−0.177	0.002
**WBPQ**	Sleep Quality	Positive well-being	0.357	<0.001
		Flourishing	0.343	<0.001
		Physical Health	0.274	<0.001
		Feeling Depressed	−0.332	<0.001
		Life Satisfaction	0.346	<0.001

**Table 3:** Significant associations between CATI subscales and SDQ and WBPQ items.

	Predictors	Outcomes	r-Value	*p*-Value
**SDQ**	Camouflaging	Hyperactivity	0.218	<0.001
		Conduct problems	0.192	0.190
		Emotional problems	0.321	<0.001
	Communication	Hyperactivity	0.198	<0.001
		Conduct problems	0.231	<0.001
		Prosocial problems	−0.217	<0.001
	Repetitive Behaviours	Hyperactivity	0.482	<0.001
		Conduct problems	0.193	<0.001
		Emotional problems	0.313	<0.001
	Cognitive Rigidity	Hyperactivity	0.261	<0.001
		Conduct problems	0.158	0.006
		Emotional problems	0.238	<0.001
	Sensory Sensitivity	Hyperactivity	0.398	<0.001
		Conduct problems	0.231	<0.001
		Emotional problems	0.256	<0.001
		Peer problems	−0.187	0.001
		Prosocial behaviour	−0.131	0.024
	Social Interactions	Hyperactivity	0.234	<0.001
		Emotional problems	0.261	<0.001
		Peer problems	−0.233	<0.001
**WBPQ**	Camouflaging	Flourishing	−0.105	0.070
		Feeling depressed	0.265	<0.001
	Repetitive Behaviours	Perceived stress	0.208	<0.001
		Life satisfaction	−0.206	<0.001
		Feeling anxious	0.268	<0.001
		Feeling depressed	0.263	<0.001
	Cognitive Rigidity	Negative well-being	0.260	<0.001
	Sensory Sensitivity	Negative well-being	0.280	<0.001
		Feeling depressed	0.265	<0.001

**Table 4:** Significant associations between CATI subscales as a covariate and SDQ items and WBPQ outcomes.

		Outcomes	Beta Values	*p*-Value
**SDQ**	CATI_SOC	Emotional Problems	0.720	0.014
		Peer problems	−0.590	0.006
	CATI_CAM	Emotional Problems	0.768	0.017
		Peer problems	−0.528	0.024
	CATI_REP	Hyperactivity	1.856	<0.001
		Emotional Problems	0.699	0.029
		Prosocial Behaviour	−0.501	0.019
**WBPQ**	CATI_RIG	Negative well-being	0.690	0.014
		Perceived Stress	0.549	0.023
	CATI_REP	Physical Health	−0.521	0.043
		Feeling Anxious	0.752	0.012

**Table 5:** Associations between WBQ predictors as a covariate and SDQ items and WBPQ outcomes.

	Predictors	Outcomes	Beta Value	*p*-Value
**SDQ**	Social Support	Peer problems	−0.226	<0.001
	Negative Coping	Hyperactivity	0.211	0.003
		Emotional problems	0.232	<0.001
		Peer problems	0.100	0.037
	Resilience	Peer problems	0.197	<0.001
		Prosocial behaviour	0.196	<0.001
	Psychological Capacity	Emotional problems	−0.334	0.003
	Student Stressors	Emotional problems	0.319	<0.001
		Prosocial behaviour	0.177	<0.001
**WBPQ**	Social Support	Positive well-being	0.212	0.001
		Flourishing	0.181	<0.001
		Physical Health	0.198	0.002
		Life Satisfaction	0.187	<0.001
		Feeling depressed	−0.329	<0.001
	Negative Coping	Negative well-being	0.208	<0.001
		Perceived stress	0.162	<0.001
		Feeling anxious	0.204	<0.001
		Feeling depressed	0.257	<0.001
	Resilience	Negative well-being	0.135	0.041
		Physical health	0.151	0.015
		Feeling depressed	0.159	0.025
	Psychological Capacity	Positive well-being	0.367	<0.001
		Negative well-being	−0.282	0.001
		Flourishing	0.436	<0.001
		Physical health	0.174	0.030
		Life satisfaction	0.304	<0.001
		Perceived stress	−0.171	0.005
		Feeling anxious	−0.294	<0.001
		Feeling depressed	−0.373	<0.001
	Student Stressors	Negative well-being	0.220	0.001
		Flourishing	−0.115	0.010
		Perceived stress	0.273	<0.001
		Feeling anxious	0.264	<0.001
		Feeling depressed	0.249	<0.001
	Academic Stress	Negative well-being	0.238	<0.001
		Flourishing	−0.099	0.026
		Perceived Stress	0.318	<0.001
		Feeling anxious	0.190	0.002
	Flow	Flourishing	0.265	<0.001
		Life satisfaction	0.185	<0.001

**Table 6:** Significant associations between sleep questions as a covariate and SDQ items and WBPQ outcomes.

	Predictors	Outcomes	Beta Value	*p*-Value
**SDQ**	Difficulty Sleeping	Hyperactivity	0.164	0.009
		Emotional problems	0.167	0.005
	Sleep Quality	Peer problems	0.213	0.002
	Daytime Sleepiness	Hyperactivity	0.236	<0.001
		Emotional problems	0.356	<0.001
**WBPQ**	Difficulty Sleeping	Perceived stress	0.132	0.006
		Feeling anxious	0.125	0.020
		Feeling depressed	0.277	<0.001
	Sleep Quality	Positive well-being	0.359	<0.001
		Flourishing	0.464	<0.001
		Physical health	0.204	0.004
		Life satisfaction	0.399	<0.001
		Perceived stress	−0.166	0.012
		Feeling anxious	−0.205	0.006
		Feeling depressed	−0.226	0.008
	Daytime Sleepiness	Negative well-being	0.316	<0.001
		Flourishing	−0.106	0.013
		Perceived stress	0.247	<0.001
		Feeling anxious	0.284	<0.001
		Feeling depressed	0.242	<0.001

**Table 7:** Associations between sleep questions, well-being predictors, and CATI subscales and SDQ items.

Scales	Predictors	Outcomes	Beta Values	*p*-Value
**Sleep Questions**	Daytime Sleepiness	Emotional problems	0.179	<0.001
**WBPQ**	Social Support	Peer problems	0.223	<0.001
	Negative Coping	Emotional problems	0.151	0.012
		Peer problems	0.115	0.028
	Resilience	Peer problems	0.174	0.005
		Prosocial behaviour	−0.160	0.006
	Psychological Capacity	Emotional problems	−0.338	<0.001
	Student Stressors	Emotional problems	0.310	<0.001
		Prosocial behaviour	−0.141	0.013
**Autistic Traits**	CATI_REP	Hyperactivity	1.762	<0.001

**Table 8:** Associations between sleep questions, well-being predictors, and CATI subscales and WBPQ outcomes.

	Outcomes	Beta Values	*p*-Value
**Difficulty Sleeping**	Feeling depressed	0.203	<0.001
**Sleep Quality**	Flourishing	0.152	0.004
	Life satisfaction	0.154	0.006
**Daytime Sleepiness**	Negative well-being	0.211	<0.001
	Perceived stress	0.131	<0.001
	Feeling anxious	0.171	<0.001
	Feeling depressed	0.130	0.013
**Social Support**	Positive well-being	0.214	0.003
	Flourishing	0.201	<0.001
	Physical health	0.177	0.017
	Life satisfaction	0.170	0.001
	Feeling depressed	−0.260	<0.001
**Negative Coping**	Negative well-being	0.141	0.004
	Flourishing	−0.112	0.005
	Perceived stress	0.137	0.002
	Feeling anxious	0.179	0.001
	Feeling depressed	0.215	<0.001
**Resilience**	Physical health	0.195	0.005
	Life satisfaction	0.119	0.017
**Psychological Capacity**	Positive well-being	0.304	<0.001
	Negative well-being	−0.211	0.016
	Flourishing	0.388	<0.001
	Life satisfaction	0.283	<0.001
	Feeling anxious	−0.236	0.004
	Feeling depressed	−0.318	<0.001
**Student Stressors**	Negative well-being	0.215	0.002
	Flourishing	−0.136	0.003
	Perceived stress	0.265	<0.001
	Feeling anxious	0.253	<0.001
	Feeling depressed	0.217	0.004
**Academic Stress**	Negative well-being	0.173	0.012
	Flourishing	−0.094	0.040
	Perceived stress	0.248	<0.001
	Life satisfaction	0.097	0.047
**Flow**	Flourishing	0.241	<0.001
	Life satisfaction	0.165	<0.001
**Rumination**	Positive well-being	0.144	0.004
**CATI_RIG**	Negative well-being	0.672	0.006

**Table 9:** Associations between CATI subscales and WBPQ predictors.

CATI Subscale	WBPQ Predictors	Beta Value	*p*-Value
**CATI_COM**	Resilience	−0.102	0.010
	Work–Life Balance	−0.097	0.031
**CATI_CAM**	Social Support	−1.105	<0.001
	Positive Coping	−0.116	<0.001
	Negative Coping	0.053	0.042
	Resilience	−0.071	0.003
	Psychological Capacity	−0.060	0.007
	Rumination	0.055	0.040
**CATI_RIG**	Social Support	0.077	0.001
	Positive Coping	0.051	0.039
	Flow	0.058	0.016

## Data Availability

Due to the sensitivity of the data, the raw data supporting the conclusions of this article will be made available by the authors upon request. Please Contact Professor Smith.

## Appendix A. Smith Short-Form Sleep Questionnaire

1. On average, how many hours of sleep do you get? _________
2. How many times do you wake up during the night? _______
3. How often do you have difficulty going to sleep? 1 = Never to 10 = very frequently.
4. How often do you wake up before you intend to? 1=Never to 10 = very frequently.
5. How would you rate the quality of your sleep? 1 = Extremely poor to 10 = very good.
6. How often do you feel sleepy during the day? 1 = Never to 10 = very often.

## Appendix B. Short-Form Student Well-Being Questionnaire

1. Year of study:
2. Gender: Male [1] Female [2].
3. Age:

Please answer the following questions about how you have felt and behaved in the last six weeks:

4. I have been experiencing positive feelings (e.g., feeling happy, satisfied with life, in good spirits, feeling good about relationships, being able to relax, and feeling energetic and interested).
5. I have been experiencing negative feelings (e.g., feeling stressed, feeling anxious or depressed, feeling physically or mentally tired, and feeling emotionally drained).
6. I feel that I have the social support I need (e.g., people to talk to, support for financial needs, friendship, and someone to discuss problems with).
7. When in a stressful situation, I try to solve the problem or seek support from others.
8. When in a stressful situation, I blame myself or wish for things to improve or avoid the problem.
9. How resilient are you (can cope and recover from adverse events)?
10. I am optimistic and confident in my problem-solving ability and generally satisfied with myself.
11. To what extent do you feel you are thriving or flourishing (e.g., being successful, feeling that life is going well, and having a sense of belonging)?
12. I have had stressful experiences (e.g., time pressure, academic dissatisfaction, loneliness, and friendship problems).
13. Do you have a high workload that makes you feel stressed and could affect how efficiently you do your work?
14. Does life outside of school interfere with your schoolwork, and does school interfere with other aspects of your life?
15. To what extent do you feel immersed in your academic work and fully engaged in your studies?
16. If you think about university work in your free time, does it have a negative effect (e.g., it makes you tense and troubled), or does it help solve problems?
17. In general, how would you rate your physical health?
18. To what extent have you been feeling stressed?
19. How satisfied are you with life?
20. To what extent have you been feeling anxious?
21. To what extent have you been feeling depressed?

## Appendix C. Short-Form Strengths and Difficulties Questionnaire

All responded to on a 10-point scale—1 = Not at all to 10 = Very much so

1. To what extent are you hyperactive and impulsive and have problems with hyperactivity (restless, fidgeting, easily distracted, acting before thinking, not finishing things)?
2. To what extent do you get angry, are disobedient, fight with others, tell lies or cheat, and take things that are not yours?
3. To what extent do you have emotional problems (worry a lot, often unhappy or downhearted, nervous in new situations, and easily scared)?
4. Are you generally liked by others of your age (you have good friends, and you are not often alone or get picked on by others)?
5. To what extent do you consider other people’s feelings, share with others, be kind to other people, and be helpful if someone is hurt?

## Appendix D. Comprehensive Autistic Traits Inventory

1. I often find myself fiddling or playing repetitively with objects (e.g., clicking pens).
2. I like to stick to certain routines for everyday tasks.
3. I expend a lot of mental energy trying to fit in with others.
4. I am over-sensitive to bright lighting.
5. There are certain activities that I always choose to do the same way every time.
6. Sometimes I watch people interacting and try to copy them when I need to socialise.
7. I often rock when sitting in a chair.
8. I generally enjoy social events.
9. I look for strategies and ways to appear more sociable.
10. In social situations, I try to avoid interactions with other people.
11. There are times when I feel that my senses are overloaded.
12. There are certain objects that I fiddle or play with that can help me calm down or collect my thoughts.
13. Reading non-verbal cues (e.g., facial expressions and body language) is difficult for me.
14. I like my belongings to be sorted in certain ways and will spend time making sure they are that way.
15. Social interaction is easy for me.
16. When interacting with other people, I spend a lot of effort monitoring how I am coming across.
17. I find social interactions stressful.
18. I am over-sensitive to touch.
19. I can tell how people feel from their facial expressions.
20. I have a tendency to pace or move around in a repetitive path.
21. I feel discomfort when prevented from completing a particular routine.
22. I rely on a set of scripts when I talk with people.
23. I find it easy to sense what someone else is feeling.
24. I am over-sensitive to particular tastes (e.g., salty, sour, spicy, or sweet).
25. I engage in certain repetitive actions when I feel stressed.
26. I rarely use non-verbal cues in my interactions with others.
27. I often insist on doing things in a certain way or re-doing things until they are ‘just right’.
28. I feel confident or capable when meeting new people.
29. Before engaging in a social situation, I will create a script to follow where possible.
30. Social occasions are often challenging for me.
31. Sometimes, the presence of a smell makes it hard for me to focus on anything else.
32. There are certain repetitive actions that others consider to be ‘characteristic’ of me (e.g., stroking my hair).
33. Metaphors or ‘figures of speech’ often confuse me.
34. It annoys me when the plans I have made are changed.
35. I find it difficult to make new friends.
36. I react poorly to unexpected loud noises.
37. I have difficulty understanding someone else’s point of view.
38. I like to arrange items in rows or patterns.
39. I try to follow certain ‘rules’ in order to get by in social situations.
40. I am sensitive to flickering lights.
41. I have certain habits that I find difficult to stop (e.g., biting/tearing nails, pulling strands of hair).
42. I have difficulty understanding the ‘unspoken rules’ of social situations.

## Appendix E. Descriptive Statistics for WBPQ Items

	**N**	**Mean**	**Std. Error**	**Std. Deviation**
**Positive Well-being**	304	5.97	0.116	2.025
**Negative Well-being**	306	6.38	0.121	2.110
**Social Support**	302	7.18	0.106	1.842
**Positive Coping**	303	6.79	0.109	1.900
**Negative Coping**	303	6.03	0.121	2.108
**Resilience**	304	6.66	0.107	1.870
**Psychological Capacity**	302	6.16	0.101	1.761
**Flourishing**	304	5.77	0.105	1.832
**Student Stressors**	300	7.65	0.103	1.789
**Academic Workload**	303	7.07	0.107	1.864
**Work Life**	304	6.67	0.122	2.126
**Flow**	304	6.07	0.100	1.747
**Rumination**	304	4.88	0.123	2.138
**Physical Health**	304	5.65	0.100	1.751
**Perceived Stress**	302	7.12	0.104	1.813
**Life Satisfaction**	302	6.39	0.093	1.614
**Feeling Anxious**	303	6.87	0.119	2.077
**Feeling Depressed**	304	4.98	0.138	2.404

## Appendix F. Descriptive Statistics for SDQ Items

	**N**	**Mean**	**Std. Error**	**Std. Deviation**
**Hyperactivity**	303	5.78	0.132	2.289
**Conduct problems**	304	2.92	0.108	1.879
**Emotional Problems**	302	6.38	0.132	2.302
**Peer problems**	303	7.31	0.093	1.618
**Prosocial problems**	303	8.17	0.084	1.469

## Appendix G. Descriptive Statistics for Sleep Questions

	**N**	**Mean**	**Std. Error**	**Std. Deviation**
**Hours of sleep**	304	2.19	0.025	0.436
**Nighttime wakefulness**	303	1.31	0.029	0.499
**Difficulty sleeping**	304	5.54	0.141	2.452
**Early Wakefulness**	302	4.60	0.141	2.446
**Sleep Quality**	303	6.12	0.096	1.677
**Daytime Sleepiness**	304	6.92	0.136	2.368

## Appendix H. Descriptive Statistics for CATI Subscales

	**N**	**Mean**	**Std. Error**	**Std. Deviation**
**CATI_SOC**	301	21.32	0.133	2.301
**CATI_COM**	302	17.88	0.202	3.515
**CATI_CAM**	300	19.60	0.371	6.419
**CATI_RIG**	300	22.52	0.344	5.961
**CATI_REP**	303	20.53	0.387	6.736
**CATI_SEN**	297	19.00	0.393	6.771
